# Effect of Primary Dysmenorrhea on the Mental Health Quality of Medical Students in Saudi Arabia

**DOI:** 10.7759/cureus.102827

**Published:** 2026-02-02

**Authors:** Sumayah Aljhani, Yaqeen Fahad Alrubaish, Thekra Abdulrahman Alsamel, Latifah Yousef Almutlaq, Summer Abdulrahman Alkhomairi, Majd Sulaiman ALsaqabi, Raghad Ibrahim Albarrak

**Affiliations:** 1 Department of Psychiatry, College of Medicine, Qassim University, Buraydah, SAU; 2 Department of Family Medicine, Family Medicine Academy, Buraydah, SAU; 3 Department of Obstetrics and Gynecology, King Fahad Medical City, Riyadh, SAU; 4 Department of Obstetrics and Gynecology, Maternity and Children's Hospital, Buraydah, SAU; 5 Department of General Surgery, King Fahad Specialist Hospital, Buraydah, SAU

**Keywords:** dysmenorrhea, menstrual pain, mental health, psychological distress, quality of life, surveys and questionnaires

## Abstract

Objective: Primary dysmenorrhea is an underdiagnosed and ignored severe lower abdominal pain that causes millions of women worldwide to suffer. This commonly affects women before the start of their period and can last for three days from its onset. Studies in Saudi Arabia have mentioned that the severity of primary dysmenorrhea can cause significant disturbances and negatively impact the lives of women, especially students. This study aimed to assess the association between primary dysmenorrhea and psychological distress among medical students at Qassim University, Qassim Region, Saudi Arabia.

Methods: This cross-sectional study was conducted among medical students from Qassim University, Saudi Arabia. A self-administered questionnaire was distributed online to medical students. The questionnaire included sociodemographic characteristics (e.g., age and marital status), menstrual characteristics (i.e., age at menarche and menstrual pain), the Dysmenorrhea Symptom Interference (DSI) scale, and the Mental Health Quality of Life (MHQoL) questionnaire.

Results: Among the 194 medical students who participated in the study, 97.9% were single and 51.5% were aged between 21 and 23 years; 25.8% experienced severe to very severe menstrual pain. The DSI scale was inversely correlated with the MHQoL. Higher DSI scores were associated with frequent or severe to very severe menstrual pain, the use of painkillers and herbs for menstrual pain, and visiting a doctor for menstrual pain. In contrast, higher MHQoL scores were associated with infrequent or mild to moderate menstrual pain.

Conclusion: Primary dysmenorrhea adversely affects the mental health of female medical students. The burden of menstrual pain, particularly in its severe form, increases the risk of mental health impairment in this group of patients. We advocate the promotion of health education programs to limit the effects of dysmenorrhea on students’ quality of life.

## Introduction

Dysmenorrhea is one of the most predominant complaints in both pre-adult and adult women, which begins just before or at the commencement of menses in the absence of any pelvic etiologies [1]. It is described as spasmodic and agonizing pain within the lower abdomen that can radiate to the back or thighs. It is classified into primary and secondary dysmenorrhea. In primary dysmenorrhea (PD), no underlying pathology can be identified, while secondary dysmenorrhea is related to recognized pathologies such as endometriosis and fibroids [2]. A significant proportion of women experience prevalent but often overlooked health issues, such as dysmenorrhea, premenstrual syndrome (PMS), and premenstrual dysphoric disorder (PMDD). A recent systematic review found primary dysmenorrhea to be highly prevalent in Saudi Arabia, ranging from 60.9% to 93.3% [3]. Previous studies have attempted to investigate the frequency, significance, and contributing variables of dysmenorrhea, PMS, and PMDD, especially in particular populations such as medical students [4-8]. Dysmenorrhea is found to affect quality of life and is associated with various physical symptoms such as headache, joint pain, nausea, vomiting, constipation, and excessive sweating [1]. Apart from physical symptoms and their impact on quality of life, previous studies have shown that the severity of dysmenorrhea is linked to various psychological disorders, including depression [9], anxiety, psychological distress [10], and attention deficit hyperactivity disorder [11]. Although menstruation occurs for only a few days, distress related to dysmenorrhea may persist between menstrual cycles.

Despite their pervasiveness, conditions such as dysmenorrhea, PMS, and PMDD are not well understood or known to the general public. The insufficient comprehension of symptoms associated with menstruation, coupled with the psychological distress they induce, not only hinders their prompt identification and treatment, but also reinforces the misconception that such symptoms are inconsequential or inherent aspects of female biology [2,3].

To address the unique needs of this population and create an environment that supports both academic success and personal well-being, medical schools, support services, and healthcare interventions can benefit from an understanding of the prevalence and impact of these conditions among medical students and determine whether there is a difference in the level of knowledge and prevalence [12,13]. Although previous studies have assessed factors related to dysmenorrhea, we found that studies assessing mental health quality of life in relation to dysmenorrhea are lacking; therefore, the aim of this research was to clarify the impact of these conditions on people’s mental health quality of life.

## Materials and methods

Survey participants and questionnaire

This study followed a descriptive cross-sectional design and used a questionnaire distributed among female medical students at Qassim University. The calculated sample size was 191 at 95% confidence interval and ± 5 margin of error. One hundred and ninety-four responses were collected over a period of two months using convenience sampling. The study eligibility criteria explicitly included participants with primary dysmenorrhea, thereby minimizing physiological and psychological confounding factors associated with underlying gynecological diseases. This approach ensured that the observed effects on mental health-related quality of life were attributable specifically to primary dysmenorrhea in young female participants.

The questionnaire comprised three parts. The first part was adopted and modified from a previous study on sociodemographic information related to the menstrual cycle (Appendix 1) [11]. The first part included age, marital status, giving birth, family monthly income, academic level, grade point average, history of psychological disorders, and age of menarche. Variables related to the menstrual cycle, including regularity, menstrual cycle length, menstrual pain duration, location, severity, and affecting working ability, in addition to using analgesics, herbs, or needing medical care. The history of gynecological diseases, abdominal surgeries, and the use of intrauterine contraception were included as well. 

The second part was the Dysmenorrhea Symptom Interference (DSI) scale (Appendix 2). The DSI scale is a 5-point self-administered Likert-type scale ranging from 1: “Not at all” to 5: “Very much,” composed of nine items and with a total score of 1 to 5, with higher scores indicating higher interference. Permission was obtained from the MAPI Research Trust [14].

The third section included the Mental Health Quality of Life (MHQoL) Questionnaire (Appendix 3). The MHQoL is a self-administered descriptive questionnaire that assesses seven dimensions: self-image, independence, mood, relationships, daily activities, physical health, and future, each with four responses ranging from very satisfied (3) to very dissatisfied (0). The scores range from 0 to 21, with higher scores indicating a better quality of life. In addition to the visual analog scale, psychosocial well-being is scored on the range from 0 (“worst imaginable psychological well-being”) to 10 (“best imaginable psychological well-being “) [15].

Ethical consideration

The Committee of Research Ethics at Qassim University approved this study with approval number 24-06-09. The participants were informed about the purpose of the study and confidentiality; electronic written consent was obtained before proceeding to the questionnaire.

Statistical analysis

Descriptive statistics are presented as numbers and percentages (%) for all categorical variables. Continuous variables were calculated and summarized as means, standard deviations, and medians (min-max). The differences in the DSI and MHQoL scores according to the sociodemographic and menstrual characteristics of the medical students were assessed using the Mann-Whitney Z-test and Kruskal-Wallis H-test. Spearman’s correlation coefficient was also calculated to determine the correlation between the DSI scale and MHQoL scores. Normality tests were performed using the Shapiro-Wilk test and the Kolmogorov-Smirnov test. Both the DSI scale and MHQoL scores followed a non-normal distribution. Therefore, nonparametric tests were applied. Statistical significance was set at P < .05. Data were analyzed using the software program IBM SPSS Statistics for Windows, Version 26 (Released 2019; IBM Corp., Armonk, New York, United States).

## Results

A total of 194 female medical students were enrolled in this study. Table 1 presents the sociodemographic characteristics of the medical students. A total of 100 (51.5%) of the patients were aged between 21 and 23 years. Most of the students were single (190; 97.09%) and had no children (190; 97.09%). An adequate monthly income was indicated by 92 (47.4%) of the respondents. Students in their fifth academic year constituted 43 (22.01%). In addition, 85 (43.08%) of patients had a GPA between 4.5 and 5.0. A total of 44 (22.07%) of the participants had a previous diagnosis of a psychological disorder.

**Table 1:** Sociodemographic characteristics of the medical students (n=194)

Study variables	N (%)
Age group	
18 – 20 years	36 (18.06%)
21 – 23 years	100 (51.50%)
24-28 years	58 (29.09%)
Marital status	
Single	190 (97.09%)
Married	04 (02.01%)
With children	
Yes	04 (02.01%)
No	190 (97.09%)
Family's monthly income status	
Enough	92 (47.04%)
Enough with savings	89 (45.09%)
Not enough	13 (06.07%)
Academic year level	
First Year	17 (08.08%)
Second Year	26 (13.04%)
Third Year	36 (18.06%)
Fourth Year	31 (16.00%)
Fifth Year	43 (22.01%)
Internship	41 (21.01%)
Last GPA	
2 - <2.75	04 (02.01%)
2.75 – 3.5	27 (13.09%)
3.75 – 4.0	78 (40.02%)
4.5 – 5.0	85 (43.08%)
Have you ever been diagnosed with any psychological disorder?	
Yes	44 (22.07%)
No	150 (77.03%)

**Figure 1:**
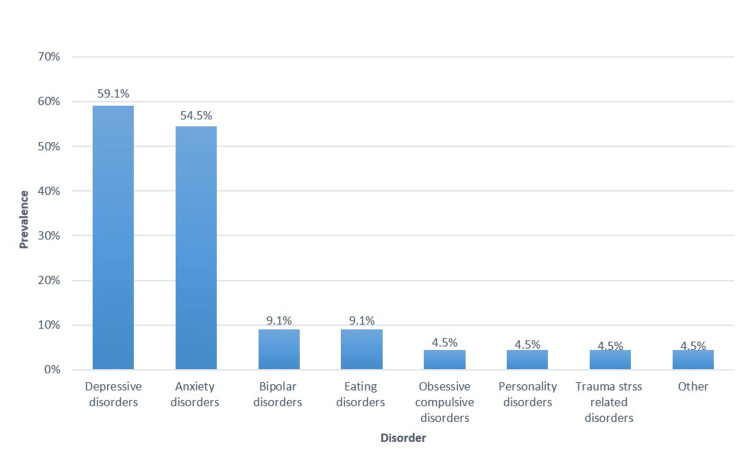
Specific diagnosis of psychological disorders among female medical students (N = 144)

Among the medical students diagnosed with psychological disorders (N = 44), the most common were depressive disorders (59.1%), anxiety disorders (54.5%), and bipolar disorders (9.1%) (Figure 1).

Regarding the menstrual characteristics of medical students (Table 2), 131 (67.05%) experienced their first menstrual cycle at the age of 12 years or younger. Most students reported regular menstrual cycles 159 (82%), with 28 days or fewer being the most common duration of menstruation (113; 58.02%). A total of 63 (32.05%) of participants reported that they were currently menstruating. The percentage of students who indicated that menstrual pain affected their working ability on a regular basis was 21 (10.08%), with the inguinal region being the most common location of pain, 128 (66%). Furthermore, 36 (18.06%) of the students experienced severe pain for approximately 1 to 2 days 128 (66%). The percentage of students who used analgesics for menstrual pain was 115 (59.03%). The use of traditional medicine for menstrual pain was reported to be 87 (44.08%). Only 34 (17.5%) of the patients visited a doctor because of menstrual pain. The proportion of medical students diagnosed with gynecological diseases was 19 (9.08%). Previous use of intrauterine contraception was reported by seven patients (3.06%), while those who had undergone previous pelvic or abdominal surgeries constituted 8 (4.01%).

**Table 2:** Menstrual characteristics of the medical students (n=194) *Some participants have multiple menstrual pain locations.

Characteristics	N (%)
Age at menarche	
≤12 years	131 (67.05%)
>12 years	63 (32.05%)
Regular menstrual cycle	
Yes	159 (82.00%)
No	35 (18.00%)
Duration of menstrual cycle	
≤28 days	113 (58.02%)
>28 days	62 (32.00%)
Not specified	19 (09.08%)
At which stage are you currently?	
Currently menstruating	63 (32.04%)
Within one week before menses	50 (25.08%)
Neither	81 (41.08%)
How much does menstrual pain affect your working ability?	
Never	07 (03.06%)
Rarely	52 (26.08%)
Sometimes	75 (38.07%)
Often	39 (20.01%)
Always	21 (10.08%)
Menstrual pain location *	
Not painful	14 (07.02%)
Lumbar region (lower back)	106 (54.06%)
Thigh region	80 (41.02%)
Inguinal region (lower abdomen)	128 (66.00%)
Menstrual pain severity	
No pain	14 (07.02%)
Mild	43 (22.02%)
Moderate	87 (44.08%)
Severe	36 (18.06%)
Very Severe	14 (07.02%)
Days of menstrual pain	
No menstrual pain	11 (05.06%)
1 – 2 days	128 (66.00%)
3 – 4 days	19 (09.08%)
5 or more days	36 (18.06%)
Do you use any painkillers for menstrual pain?	
Yes	115 (59.03%)
No	79 (40.07%)
Do you use any herbs for menstrual pain?	
Yes	87 (44.08%)
No	107 (55.02%)
Have you ever been to a doctor because of menstrual pain?	
Yes	34 (17.05%)
No	160 (82.05%)
Have you ever been diagnosed with any gynecological disease?	
Yes	19 (09.08%)
No	175 (90.02%)
Have you been using intrauterine contraception	
Yes	07 (03.06%)
No	187 (96.04%)
Have you had pelvic or abdominal surgeries?	
Yes	08 (04.01%)
No	186 (95.09%)

**Figure 2:**
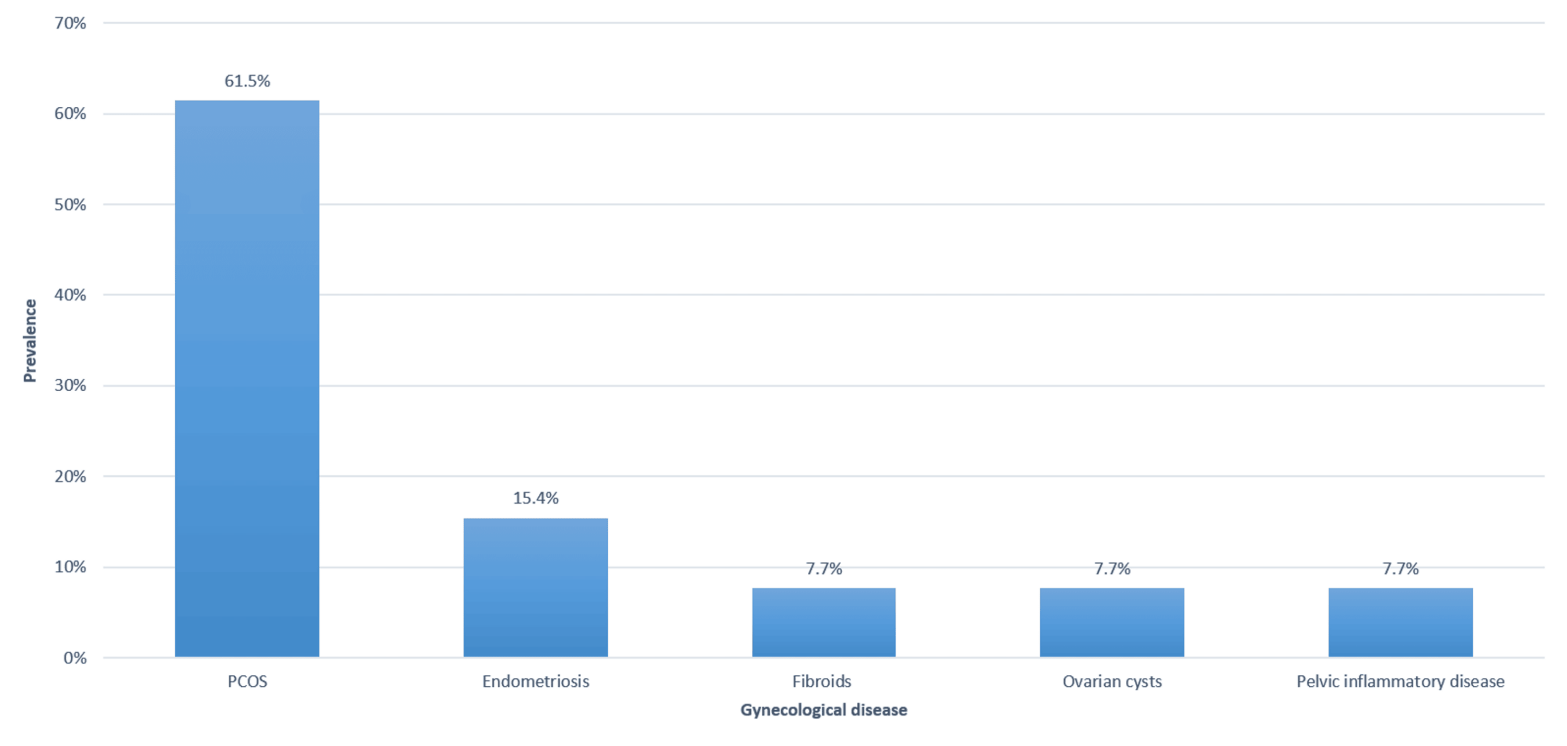
Specific diagnosis of gynecological diseases among female medical students (N = 34)

Among the students diagnosed with gynecological disorders (n = 34), the most commonly diagnosed gynecological diseases were PCOS (61.5%) and endometriosis (15.4%) (Figure 2).

Regarding the descriptive statistics of the DSI scale and MHQoL questionnaire, the mean DSI scale score was 2.59 (SD = 1.08), with a mean percentage score of 51.80%. For the MHQoL, the mean score was 12.6 (SD = 4.29), and the mean percentage score was 60% (Table 3).

**Table 3:** Descriptive statistics of the DSI scale and MHQoL-7D questionnaire (n=194) DSI: Dysmenorrhea Symptom Interference; MHQoL: Mental Health Quality of Life.

Variable	Mean ± SD	Mean (%)	Median (min-max)
DSI	2.59 ± 1.08	51.80%	2.33 (1.00 – 5.00)
MHQoL	12.6 ± 4.29	60.00%	13.0 (1.00 – 21.0)

**Figure 3:**
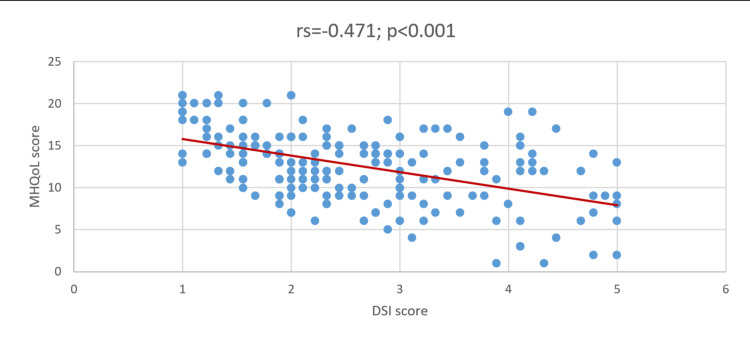
Correlation between DSI and MHQoL DSI: Dysmenorrhea Symptom Interference; MHQoL: Mental Health Quality of Life.

As shown in Figure 3, the Spearman-Rho correlation coefficient indicated an inverse correlation between the DSI and MHQoL scores (rs = 0.471; P < .001).

Exploring the differences in DSI and MHQoL in terms of the sociodemographic characteristics of the medical students (Table 4), we found that only the previous diagnosis of psychological disorder showed significant differences in MHQoL scores, with students who had no previous mental disorders showing significantly higher MHQoL scores (Z = 2.476; P = .013). Other sociodemographic characteristics, including age group, monthly family income, academic year level, and GPA, were not significantly different between the DSI and MHQoL groups (P > .05).

**Table 4:** Differences in DSI and MHQoL scores in relation to the socio-demographic characteristics of the medical students (n=194) ^a ^P-value has been calculated using the Mann-Whitney Z-test. ^b ^P-value has been calculated using the Kruskal-Wallis H-test. **Significant at p<.05 level. DSI: Dysmenorrhea Symptom Interference; MHQoL: Mental Health Quality of Life.

Factor	DSI Score (5) Mean ± SD	H/Z-test; P-value	MHQoL Score (5) Mean ± SD	H/Z-test; P-value
Age group ^a^				
· ≤22 years	2.54 ± 1.14	Z=1.141; P=0.254	12.9 ± 4.28	Z=0.545; P=0.586
· >22 years	2.64 ± 1.01		12.4 ± 4.32	
Family's monthly income status ^b^				
· Enough	2.69 ± 1.09	H=1.327; P=0.515	12.7 ± 4.02	H=1.700; P=0.427
· Enough with savings	2.50 ± 1.07		12.8 ± 4.62	
· Not enough	2.51 ± 0.97		11.0 ± 3.94	
Academic year level ^b^				
· Basic science student (1^st^-3^rd^ year)	2.59 ± 1.21	H=0.614; P=0.736	12.7 ± 4.47	H=0.072; P=0.965
· Clinical science student (4^th^ – 5^th^ year)	2.56 ± 1.02		12.6 ± 4.27	
· Internship	2.63 ± 0.89		12.5 ± 4.11	
Last GPA ^b^				
· ≤3.5	2.53 ± 1.26	H=0.728; P=0.695	12.2 ± 5.39	H=1.041; P=0.594
· 3.75 – 4.0	2.57 ± 0.99		12.4 ± 4.19	
· 4.5 – 5.0	2.63 ± 1.08		13.0 ± 3.97	
Diagnosed with a psychological disorder ^a^				
· Yes	2.61 ± 0.96	Z=0.509; P=0.611	11.3 ± 4.57	Z=2.476; P=0.013 **
· No	2.58 ± 1.11		13.0 ± 4.15	

When measuring the differences in DSI and MHQoL scores in relation to the menstrual characteristics of the medical students (Table 5), it was observed that a higher DSI score was associated with experiencing menstrual pain often or always affecting working ability (Z = 7.540; P < .001), experiencing severe to very severe pain during menstruation (Z = 5.478; P < .001), use of painkillers for menstrual pain (Z = 5.109; P < .001), use of herbs (Z = 2.681; P = .007) and doctor visit due to menstrual pain (Z = 2.085; P = .037). In contrast, higher MHQoL scores were more associated with rare or occasional menstrual pain (Z = 3.007; P = .003) and mild-to-moderate menstrual pain severity (Z = 2.546; P = .011).

**Table 5:** Differences in DSI and MHQoL scores according to the menstrual characteristics of the medical students (n=194) § P-value has been calculated using the Mann-Whitney Z-test. ** Significant at p<.05 level. DSI: Dysmenorrhea Symptom Interference; MHQoL: Mental Health Quality of Life.

Factor	DSI Score (5) Mean ± SD	Z-test; P-value §	MHQoL Score (5) Mean ± SD	Z-test; P-value §
Age at menarche				
≤12 years	2.49 ± 1.05	Z=1.826; P=0.068	12.5 ± 4.45	Z=1.018; 0.309
>12 years	2.79 ± 1.11		13.0 ± 3.97	
Regular menstrual cycle				
Yes	2.63 ± 1.09	Z=1.158; P=0.247	12.5 ± 4.47	Z=0.682; P=0.495
No	2.41 ± 0.97		13.3 ± 3.42	
Duration of menstrual cycle				
≤28 days	2.63 ± 1.12	Z=0.164; P=0.870	12.5 ± 4.46	Z=0.732; P=0.464
>28 days	2.64 ± 1.02		13.0 ± 3.91	
At which stage are you currently?				
Currently menstruating	2.37 ± 0.98	Z=1.398; P=0.162	13.2 ± 4.24	Z=1.665; P=0.096
Within one week before menses	2.71 ± 1.19		11.9 ± 4.22	
How much does menstrual pain affect your working ability?				
Rarely or sometimes	2.21 ± 0.84	Z=7.540; P<0.001 **	13.1 ± 4.06	Z=3.007; P=0.003 **
Often or always	3.51 ± 0.99		11.2 ± 4.35	
Menstrual pain severity				
Mild to moderate	2.38 ± 0.87	Z=5.478; P<0.001 **	12.9 ± 4.09	Z=2.546; P=0.011 **
Severe to very severe	3.44 ± 1.14		11.2 ± 4.41	
Days of menstrual pain				
1 – 2 days	2.55 ± 1.07	Z=1.857; P=0.063	12.8 ± 4.23	Z=0.582; P=0.561
>2 days	2.86 ± 1.05		12.1 ± 4.24	
Use of any painkillers for menstrual pain				
Yes	2.92 ± 1.13	Z=5.109; P<0.001 **	12.1 ± 4.62	Z=1.756; P=0.079
No	2.11 ± 0.77		13.4 ± 3.70	
Use of any herbs for menstrual pain				
Yes	2.84 ± 1.15	Z=2.681; P=0.007 **	12.3 ± 4.62	Z=0.794; P=0.427
No	2.39 ± 0.97		12.9 ± 4.01	
Have you ever been to a doctor because of menstrual pain?				
Yes	2.99 ± 1.26	Z=2.085; P=0.037 **	11.8 ± 4.87	Z=1.275; P=0.202
No	2.51 ± 1.02		12.8 ± 4.16	
Have you ever been diagnosed with any gynecological disease?				
Yes	2.49 ± 1.13	Z=0.538; P=0.590	12.0 ± 5.23	Z=0.462; P=0.644
No	2.60 ± 1.07		12.7 ± 4.19	

## Discussion

This study aimed to measure the effect of primary dysmenorrhea on the mental health-related quality of life in female medical students. The findings of this study may contribute to understanding the trend of dysmenorrhea in the younger female population and how it affects their mental well-being in everyday schools. The results of this study showed that the mean DSI was 2.59 out of 5 points, with a mean percentage score of 51.80%, suggesting that dysmenorrhea likely affected more than half of the students. Furthermore, approximately 93% of the students experienced mild (43; 22.02%) to very severe (14; 7.02%) menstrual pain, which affected their daily activities. Consistent with our findings, studies conducted in this region have shown that dysmenorrhea is prevalent in female students [3,4-7,11-13]. A similar prevalence of dysmenorrhea has been tagged as the leading cause of absenteeism among college students [16]. Similarly, a study conducted among Nigerian university students showed a consistent dysmenorrhea rate of 69.08% [17].

Our study revealed no significant factors associated with DSI and demographic data. A previous investigation conducted by Al-Jammaz et al. revealed findings regarding the association between PMS and demographic factors. According to previous reports [7], the age of students was a factor in the development of PMS, while BMI, family history of PMS, and smoking were insignificant; however, these variables were not tested in our study. Consistent with previous reports, Balaha et al. found a significant difference between age and PMS, with older age groups being most affected by PMS [12]. In addition, rural residence, regular menses, lower menarche age, and a family history of PMS contribute to the same effect [12]. Both studies used questionnaires guided by the American College of Obstetrics and Gynecology (ACOG) criteria to diagnose PMS, which possibly accounted for this difference. Increasing DSI scores were associated with regular menstrual pain and higher levels of pain (severe to very severe), affecting daily routines. This finding is in agreement with the results of Fathi et al. [18], which reported that discomfort and pain were significantly associated with dysmenorrhea (P < .01). Personal care has been significantly reduced as well [18]. Yilmaz and Avci [19] found that a vast majority of female students indicated that dysmenorrhea restricted their daily activities and social interactions, leading to an increase in absence from class. This could be explained by the different pain threshold level, psychological perception of pain, and its effect as well as the decreased activity level on mental health. Menstrual bleeding, menstrual patterns, and dysmenorrhea ratings are menstrual factors linked to the development of dysmenorrhea among female Turkish students [19].

The use of medications and traditional medicines to relieve menstrual pain was common in our population. The results of our study suggest that nearly 60% of students with dysmenorrhea took painkillers (P < .001), while approximately half tried herbs for menstrual pain (P = .007). Only 17.05% of women visited a doctor to seek advice on menstrual pain (P = .037). This result mirrors the findings of Alateeq et al. [11]. Severe dysmenorrhea was detected in 30.08% of the patients, requiring painkillers, herbs, or medical attention [11]. However, this finding contradicts those of Esan et al. [17], who reported that diversion therapy was the most prominent treatment choice, followed by the hot-bottle technique, lower back abdominal massage, and breathing exercises. At least one-third of the participating students used medications and herbs [17]. The choice of pharmacological and non-herbal intervention can be explained by the medical background of our sample as well as the fast need to relieve menstrual pain, which could affect the level of function, in addition to the stress imposed by high academic demands that could also alter pain sensitivity.

The total mean MHQoL score was 12.6 out of 21 points (mean percentage score, 60%), suggesting a better psychological condition in more than half of the students. However, nearly one quarter (22.07%) of our participants had a history of psychological disorders, with depressive and anxiety disorders being the most common. This is comparable with a study conducted in the UAE, with depressed mood, muscle, joint, abdominal, and back pain; feelings of anger; and craving for certain foods being the most frequently reported psychological conditions associated with PMS symptoms [13], which is consistent with inguinal pain being the most reported in our study. Poor MHQoL was associated with a previous diagnosis of psychological disorders, regular menstrual pain, and severe to very severe menstrual pain. This explains the possibility of different pain perception or preoccupation by a person with psychological disorders; vice versa, the consistency of menstrual pain and severity can negatively impact mental well-being. This agrees with the findings of Alateeq et al. [11], who found that menstrual pain severity and working ability were positively correlated with depression. Notwithstanding these reports, Lockinger & Ganon [9], this study aimed to highlight the correlation between the DSI and MHQoL. Based on the Spearman correlation coefficient, an inverse correlation between the DSI and MHQoL was observed, suggesting that an increase in the DSI score was correlated with a decrease in the MHQoL score. In other words, the greater the interference of dysmenorrhea, the worse the impairment of the patient’s mental condition. Consistent with our results, a similar pattern of association has been observed across studies, suggesting that dysmenorrhea negatively affects the psychological well-being of female students [4-6,19]. Severe dysmenorrhea has been linked to the development of depression in young schoolgirls [11]. It could also lead to reduced physical activity, poor work satisfaction, and loss of concentration [16], which in turn could negatively affect mental health. The identification of these factors is critical and may serve as intervention points.

Several potential pathways may explain the link between low-quality mental health and dysmenorrhea. One possible physiological pathway suggests that depression and other negative emotions can lead to psychological imbalances and neuroendocrine disturbances, which may stimulate the uterus and increase tension in the uterine isthmus, potentially causing or worsening dysmenorrhea. Research supports the monoamine hypothesis of depression, which indicates that serotonin release is weakened in individuals with depression and behavioral factors such as dietary habits, binge eating, and sleep quality, making them more sensitive to dysmenorrhea [20].

Additional pathways may explain the adverse effects of chronic pain on women's mental health. Excessive secretion of uterine prostaglandins, which are regulated by progesterone and estrogen, is a key factor. Hormonal fluctuations during the menstrual cycle affect emotion regulation through their impact on the brain. Estrogen and progesterone have been implicated in depressive symptoms in many women, with variations in ovarian hormone levels associated with a higher risk of depression. Elevated estrogen and prostaglandin levels are probable contributors to dysmenorrhea [21]. Apart from dysmenorrhea, which can be a stressful trigger, chronic pain has long been linked to depression, which is hypothesized to be mediated by different numbers of neurotransmitters, including norepinephrine and serotonin, which play a significant role in mental health [22]. Furthermore, primary dysmenorrhea was not only linked to depression but to stress and anxiety as well [23], as previously illustrated that the correlation can be both ways. Specifically, menstruation severity was found to be positively correlated to depression, anxiety, and stress [8], which is consistent with our finding. Additionally, this link is potentially influenced genetically as several Single-nucleotide polymorphism was found to increase the risk [24].

This study has some limitations. The sample size was limited to medical students, which limits the generalizability of the findings. Additionally, a cross-sectional study requires a large sample size to precisely represent the study population; however, this was not reflected in our population because of the limited sample size. Although the current study demonstrates an association between dysmenorrhea and mental health-related quality of life, the findings are limited by the cross-sectional nature of the study. Notably, 22.7% of participants reported a diagnosed psychological disorder, which may have contributed to functional pain or increased pain severity. Future longitudinal studies with larger and more diverse populations are recommended to further examine this relationship across the menstrual cycle. Future longitudinal studies are recommended to assess mental health-related quality of life throughout the entire menstrual cycle.

## Conclusions

Primary dysmenorrhea negatively affects the mental health of female medical students. Greater dysmenorrhea interference was observed in female students who were taking painkillers or traditional medicines and those who visited a doctor due to menstrual pain. Furthermore, female students who regularly experienced severe to very severe menstrual pain have been associated with lower mental health-related quality of life. The use of medications and herbs to treat menstrual pain was common among the students. This study supports the inclusion of health education programs for students at the university level to prevent issues associated with dysmenorrhea which limit their activities and affect their school lives. Effective management of dysmenorrhea is key to improving the quality of life of female medical students.

## Appendices

**Table 6:** Sociodemographic details Credits: Deemah Alateeq [11]

Age	(open answer)
Marital status	Married
	Single
	Divorced
	Widow
Do you have children	Yes
	No
Family’s monthly income	Enough
	Enough with savings
	Not enough
	Not enough and without savings
At what year are you currently?	First year
	Second year
	Third year
	Fourth year
	Fifth year
	Internship
What is your last GPA?	2- <2.75
	> 2.75
	> 3.75
	>4.5
Age of first menarche	(open answer)
Is your menstrual cycle regular?	Yes
	No
How long is your menstrual cycle? From the first day of your menstrual period till the next one in the next month, how many days (Ex: my period is 6 days and I have to wait 21 days till the next one = 27)	(open answer)
In which stage are you currently?	Within 1 week before menses
	Currently menstruating
	Neither
How much does menstrual pain affect your working ability?	Never
	Rarely
	Sometimes
	Often
	Always
Menstrual pain location(s) (you can choose several locations)	It is not painful
	Lumbar region (lower back)
	Thigh region
	Inguinal region (lower abdomen)
Menstrual pain severity	No pain
	Mild
	Moderate
	Severe
	Very severe
Days of menstrual pain	0 days
	1-2 days
	3-4 days
	5 days or more
Do you use any painkillers for menstrual pain?	Yes
	No
Do you use any herbs for menstrual pain?	Yes
	No
Have you ever been to a doctor because of menstrual pain?	Yes
	No
Have you ever been diagnosed with any gynecological disease?	Yes
	No
If yes, what is it?	Endometriosis
	Fibroids
	Adenomyosis
	Endometrial polyps
	Interstitial cystitis
	Pelvic inflammatory disease
	Other
Have you been using intrauterine contraception?	Yes
	No
Have you had pelvic or abdominal surgeries?	Yes
	No
Have you ever been diagnosed with any psychological disorder?	Yes
	No
If yes, what is it?	Depressive disorder
	Anxiety disorder
	Obsessive-compulsive disorder
	Bipolar disorder
	Eating disorder
	Psychotic disorder
	Personality disorder
	Trauma and stress-related disorders
	Autism
	ADHD
	Substance use disorder
	Other

**Table 7:** Dysmenorrhea Symptom Interference (DSI) scale The permission was obtained from the MAPI Research Trust [14].

During your last menstrual period, please check how much your menstrual pain and menstrual gastrointestinal (Gl) symptoms interfered with:	Not at all
	A little bit
	Some what
	Quite a bit
	Very much
Physical activities (e.g., walk, run, swim, yoga, & other exercises)	
Sleep (falling or staying asleep)	
Daily Activities	
Work (including work, housework, schoolwork, homework	
Concentration	
Enjoyment of life	
Leisure activities (time spent relaxing, having fun, doing hobbies	
Social activities (time spent with family, friends, etc.)	
Mood (irritable, anxious frustrated, depressed, etc.)	

**Table 8:** Mental health quality of life questionnaire (MHQoL) Available for use without prior permission from the authors at the Institute for Medical Technology Assessment  (https://www.imta.nl/questionnaires/mhqol/) [15]

Please indicate below which statements best describe your situation TODAY by ticking ONE box in each of the seven subjects.
Self-Image
I think very positively about myself
I think positively about myself
I think negatively about myself
I think very negatively about myself
Independence For example: freedom of choice, financial, co-decision making
I am very satisfied with my level of independence
I am satisfied with my level of independence
I am dissatisfied with my level of independence
I am very dissatisfied with my level of independence
Mood
I do not feel anxious, gloomy, or depressed
I feel a little anxious, gloomy, or depressed
I feel anxious, gloomy, or depressed
I feel very anxious, gloomy, or depressed
Relationships For example: partner, children, family, friends
I am very satisfied with my relationships
I am satisfied with my relationships
I am dissatisfied with my relationships
I am very dissatisfied with my relationships
Daily Activities For example: work, study, household, leisure activities
I am very satisfied with my daily activities
I am satisfied with my daily activities
I am dissatisfied with my daily activities
I am very dissatisfied with my daily activities
Physical Health
I have no physical health problems
I have some physical health problems
I have many physical health problems
I have a great many physical health problems
Future
I am very optimistic about my future
I am optimistic about my future
I am gloomy about my future
I am very gloomy about my future
Pyschological Well-Being
On the scale below, please indicate with an X how you rate your psychological well-being. 0 represents the worst imaginable psychological well-being, while 10 represents the best imaginable psychological well-being.
0
1
2
3
4
5
6
7
8
9
10

## References

[1] Itani R, Soubra L, Karout S, Rahme D, Karout L, Khojah HM (2022). Primary dysmenorrhea: Pathophysiology, diagnosis, and treatment updates. Korean J Fam Med.

[2] Proctor M, Farquhar C (2006). Diagnosis and management of dysmenorrhoea. BMJ.

[3] Alzebidi JA, Almohaimeed SA, Alharbi GA (2022). Prevalence of primary dysmenorrhea in Saudi Arabia - a systematic review. Indo Am J Pharm Sci.

[4] Hashim RT, Alkhalifah SS, Alsalman AA, Alfaris DM, Alhussaini MA, Qasim RS, Shaik SA (2020). Prevalence of primary dysmenorrhea and its effect on the quality of life amongst female medical students at King Saud University, Riyadh, Saudi Arabia. A cross-sectional study. Saudi Med J.

[5] Al-Shahrani AM, Miskeen E, Shroff F, Elnour S, Algahtani R, Youssry I, Ahmed S (2021). Premenstrual Syndrome and Its Impact on the Quality of Life of Female Medical Students at Bisha University, Saudi Arabia. J Multidiscip Healthc.

[6] Al-Batanony MA, Al-Nohair SF (2014). Prevalence of premenstrual syndrome and its impact on quality of life among university medical students, Al Qassim University, KSA. Public Health Res.

[7] AlJammaz HM, Al Jayani RT, Alanazi RM (2022). Prevalence and risk factors of premenstrual dysphoric disorder and premenstrual syndrome among medical students of Tabuk University. Saudi Med J Stud.

[8] Rogers SK, Ahamadeen N, Chen CX, Mosher CE, Stewart JC, Rand KL (2023). Dysmenorrhea and psychological distress: a meta-analysis. Arch Womens Ment Health.

[9] Lockinger K, Gagnon MM (2023). Dysmenorrhea and psychological wellbeing among females with attention deficit hyperactivity disorder. J Health Psychol.

[10] Abdelmoaty Goweda R, Hassan-Hussein A, Ali Alqahtani M (2020). Prevalence of sleep disorders among medical students of Umm Al-Qura University, Makkah, Kingdom of Saudi Arabia. J Public Health Res.

[11] Alateeq D, Binsuwaidan L, Alazwari L, Algarni M, Al Hussain M, Alzahrani R, Aljohani R (2022). Dysmenorrhea and depressive symptoms among female university students: a descriptive study from Saudi Arabia. Egypt J Neurol Psychiatr Neurosurg.

[12] Balaha MH, Amr MA, Saleh Al Moghannum M, Saab Al Muhaidab N (2010). The phenomenology of premenstrual syndrome in female medical students: a cross sectional study. Pan Afr Med J.

[13] Hashim MS, Obaideen AA, Jahrami HA (2019). Premenstrual syndrome is associated with dietary and lifestyle behaviors among university students: a cross-sectional study from Sharjah, UAE. Nutrients.

[14] Chen CX, Murphy T, Ofner S (2021). Development and testing of the dysmenorrhea symptom interference (DSI) scale. West J Nurs Res.

[15] van Krugten FC, Busschbach JJ, Versteegh MM, Hakkaart-van Roijen L, Brouwer WB (2022). The Mental Health Quality of Life Questionnaire (MHQoL): development and first psychometric evaluation of a new measure to assess quality of life in people with mental health problems. Qual Life Res.

[16] Joshi T, Kural M, Agrawal DP, Noor NN, Patil A (2015). Primary dysmenorrhea and its effect on quality of life in young girls. Int J Med Sci Public Health.

[17] Esan DT, Ariyo SA, Akinlolu EF (2024). Prevalence of dysmenorrhea and its effect on the quality of life of female undergraduate students in Nigeria. J Endometriosis Uterine Disord.

[18] Fathi M, Davoodi P, Semyari N (2022). Dysmenorrhea and quality of life: a cross-sectional survey among medical students. Interv Pain Med Neuromod.

[19] Yılmaz FA, Avcı D (2020). Effect of dysmenorrhea on quality of life in university students: a case-control study. Cukurova Med J.

[20] Li Y, Kang B, Zhao X, Cui X, Chen J, Wang L (2023). Association between depression and dysmenorrhea among adolescent girls: multiple mediating effects of binge eating and sleep quality. BMC Womens Health.

[21] Zhao S, Wu W, Kang R, Wang X (2021). Significant increase in depression in women with primary dysmenorrhea: a systematic review and cumulative analysis. Front Psychiatry.

[22] Marks DM, Shah MJ, Patkar AA, Masand PS, Park GY, Pae CU (2009). Serotonin-norepinephrine reuptake inhibitors for pain control: premise and promise. Curr Neuropharmacol.

[23] Bajalan Z, Moafi F, MoradiBaglooei M, Alimoradi Z (2019). Mental health and primary dysmenorrhea: a systematic review. J Psychosom Obstet Gynaecol.

[24] Jiangzhou H, Xu H, Wen Y (2025). Association between primary dysmenorrhea and mental health traits: a study based on multi-phenotype correlation network and Mendelian randomization analysis in female college students. Phenomics.

